# Plasmapheresis effectively abrogates severe liver toxicity of pegaspargase in a patient with acute lymphoblastic leukemia

**DOI:** 10.1007/s00277-024-05789-7

**Published:** 2024-05-11

**Authors:** Markus Tölle, Nicola Gökbuget, Stefan Habringer, Ulrich Keller, Stefan Schwartz

**Affiliations:** 1 Department of Nephrology and Medical Intensive Care (Campus Benjamin Franklin), Charité – Universitätsmedizin Berlin, corporate member of Freie Universität and Humboldt-Universität zu Berlin, Hindenburgdamm 30, 12203 Berlin, Germany; 2 Medizinische Klinik II, Universitätsklinikum der Johann-Wolfgang-Goethe Universität, Theodor-Stern-Kai 7, 60596 Frankfurt/Main, Germany; 3 German Cancer Consortium (DKTK), partner site Frankfurt/Mainz, a partnership between DKFZ and Johann-Wolfgang-Goethe Universität, 60596 Frankfurt/Main, Germany; 4 Department of Hematology, Oncology and Cancer Immunology (Campus Benjamin Franklin), Charité – Universitätsmedizin Berlin, corporate member of Freie Universität and Humboldt-Universität zu Berlin, Hindenburgdamm 30, 12203, Berlin, Germany; 5 German Cancer Consortium (DKTK), partner site Berlin, a partnership between DKFZ and Charité-Universitätsmedizin Berlin, 12203 Berlin, Germany

Dear Editor,

Asparaginase is an essential component in the treatment of acute lymphoblastic leukemia (ALL) and its use has been intensified in recent years in treatment protocols across all age groups [1]. Various formulations are available and among these, pegaspargase, a pegylated, *E. coli*-derived asparaginase, offers a long half-life requiring less frequent dosing together with a lower risk of antibody formation and hypersensitivity reactions [2]. However, the management of pegaspargase-related toxicities might be challenging beyond drug administration due to its pharmacokinetic properties.

A 57-year-old male with common ALL received consolidation therapy with high-dose methotrexate (days 1 and 15: 1 g/m^2^) and pegaspargase (days 2 and 16: 1,000 units/m^2^) within a trial evaluating an age-adapted treatment protocol (ClinicalTrials.gov, NCT 03480438, March 21 2018) [3]. The liver function tests (LFTs) slightly increased on day 11 (alkaline phosphatase 1.3x upper limit of normal). Upon further increase of LFTs, ursodeoxycholate was started on day 16 (250 mg qid), but LFTs rapidly increased further (maximum alkaline phosphatase: 13x upper limit of normal). In addition, a high asparaginase activity in the serum was measured on day 27 (642 U/L). Therefore, the decision was made to eliminate asparaginase from the circulation by plasmapheresis. On days 28–30, the patient underwent three sessions with a treated plasma volume of 1.0–1.2 of the calculated total plasma volume, which was replaced by an isotonic albumin solution. The result of this intervention was an immediate decrease of the asparaginase activity down to zero and a rapid and sustained normalisation of LFTs (Fig. 1). The antineoplastic treatment was resumed on day 42, which represents a delay of the scheduled therapy of only 6 days. At present, the patient is in continuous, complete molecular remission of his ALL while receiving maintenance therapy.

**Fig. 1:**
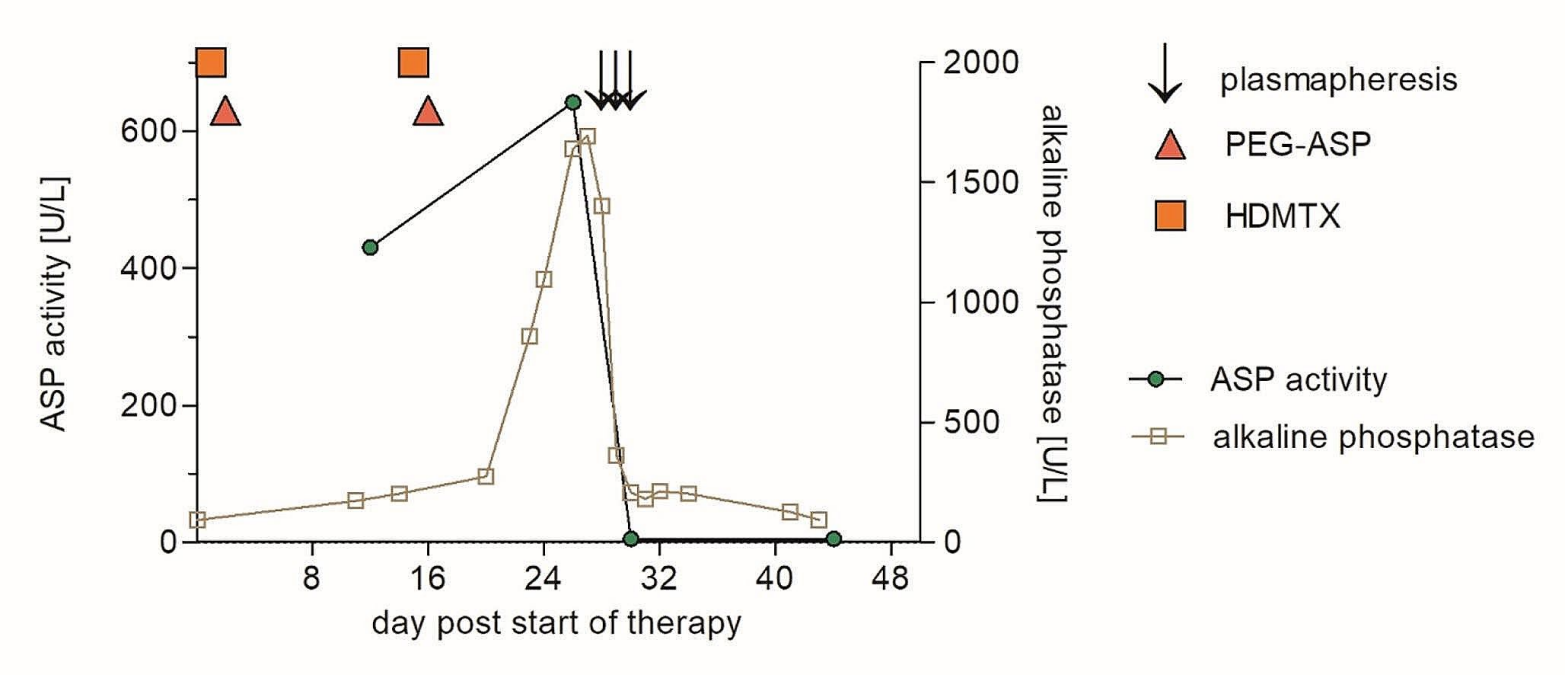
Clinical course of a patient with acute lymphoblastic leukemia and pegaspargase toxicity. Following consolidation therapy with high-dose methotrexate (HDMTX) and pegaspargase (PEG-ASP) severe liver toxicity occurred in association with high asparaginase (ASP) activity, which vanished with pegaspargase removal by plasmapheresis

Toxicities of asparaginase could affect a variety of organ systems, including the liver, and might be severe and prolonged after pegaspargase. However, extracorporeal drug removal has not been included into proposed treatment algorithms of asparaginase toxicities [4]. Only two patients treated with plasmapheresis for asparaginase liver toxicity have been reported [5, 6]. One patient had received a total dose of 3,750 units pegaspargase, whereas the other patient was treated with 5,000 units/m^2^ daily of non-pegylated L-asparaginase over 14 days. Both patients improved with plasmapheresis, but the asparaginase activity was not or incompletely monitored. Thus, it remains unclear whether the observed improvements represent a spontaneous course or were due to removal of asparaginase. In our patient, the liver toxicity correlated closely with a high asparaginase activity and rapidly vanished with asparaginase removal and decrease of the enzyme activity down to zero. We therefore advocate that plasmapheresis should be integrated into the treatment algorithms of severe asparaginase toxicities.

## Data Availability

No datasets were generated or analysed during the current study.
